# Circular RNA circLMO1 Suppresses Cervical Cancer Growth and Metastasis by Triggering miR-4291/*ACSL4*-Mediated Ferroptosis

**DOI:** 10.3389/fonc.2022.858598

**Published:** 2022-03-07

**Authors:** Rongying Ou, Shun Lu, Luhui Wang, Yebo Wang, Mingfen Lv, Tian Li, Yunsheng Xu, Jieqiang Lu, Ren-shan Ge

**Affiliations:** ^1^ Department of Obstetrics and Gynecology, The Second Affiliated Hospital and Yuying Children’s Hospital, Wenzhou Medical University, Wenzhou, China; ^2^ Department of Obstetrics and Gynecology, The First Affiliated Hospital, Wenzhou Medical University, Wenzhou, China; ^3^ Department of Dermatovenerology, The First Affiliated Hospital, Wenzhou Medical University, Wenzhou, China; ^4^ Department of Obstetrics and Gynecology, The Seventh Affiliated Hospital, Sun Yat-sen University, Shenzhen, China; ^5^ Department of Dermatovenerology, The Seventh Affiliated Hospital, Sun Yat-sen University, Shenzhen, China

**Keywords:** circular RNA, CircLMO1, MiR-4291, ferroptosis, cervical cancer

## Abstract

**Background:**

A number of studies have demonstrated that circular RNA (circRNA) plays a critical role in tumorigenesis and tumor progression. However, the biological effects of most circRNAs on cervical cancer remain unclear. Hsa_circ_0021087 (thereafter named circLMO1) is a circRNA generated from the circularization of exon 2 and exon 3 of LIM Domain Only 1 (*LMO1*) and first identified as a tumor suppressor in gastric cancer. We aimed to identify the role of circLMO1 in cervical cancer progression.

**Methods:**

CircLMO1 was verified through qPCR and Sanger sequencing. The biological role of circLMO1 in regulating cervical cancer growth and metastasis was investigated both *in vitro* and in the nude mouse xenograft tumor model. The dual luciferase reporter assay and rescue experiment were conducted to evaluate the interactions among circLMO1, microRNA (miR)-4291, and acyl-CoA synthetase long chain family member 4 (ACSL4). The role of circLMO1 in regulating ferroptosis was assessed by analyzing lipid reactive oxygen species (ROS), and malonyl dialdehyde (MDA), and glutathione (GSH) content.

**Results:**

The level of circLMO1 was down-regulated in cervical cancer tissues and was associated with the International Federation of Gynecology and Obstetrics (FIGO) staging. Functionally, circLMO1 overexpression inhibited cervical cancer growth and metastasis both *in vitro* and *in vivo*, whereas circLMO1 depletion promoted cervical cancer cell proliferation and invasion. Mechanistically, circLMO1 acted as a competing endogenous RNA (ceRNA) by sponging miR-4192 to repress target gene *ACSL4*. CircLMO1 promoted cervical cancer cell ferroptosis through up-regulating ACSL4 expression. Overexpression of miR-4291 or knockdown of ACSL4 reversed the effect of circLMO1 on facilitating ferroptosis and repressing cervical cancer cell proliferation and invasion.

**Conclusion:**

CircLMO1 acted as a tumor suppressor of cervical cancer by regulating miR-4291/*ACSL4*-mediated ferroptosis, and could be a promising biomarker for the clinical management of cervical cancer.

## Introduction

Cervical carcinoma is the second most common gynecological carcinoma after breast cancer in the world (1), with nearly 570 000 new cases and 311 000 deaths in 2018 (2). It is well known that human papillomavirus (HPV), particularly HPV16 and HPV18, is the main etiological factor of cervical carcinoma (3, 4). Although HPV vaccines are effective against HPV-related cervical cancer, and early screening can reduce the mortality of cervical cancer, most patients are already at an advanced stage at the time of diagnosis (5). For metastatic cervical cancer, the prognosis is poor, with a mean survival period of only eight to thirteen months (6). There is an urgent need to identify new functional molecules for effective early screening and treatment of cervical cancer.

Circular RNA (circRNA) is a class of evolutionarily conserved single-stranded RNA transcript, which formed by reverse splicing into a covalently closed loop (7). Unlike linear RNA, circRNA does not have a 5′ Cap or 3′ polyadenylation tail (8). Although circRNAs cannot encode proteins, they play a vital role in a variety of physiological processes such as cell differentiation (9), proliferation (10), apoptosis (11), autophagy (12), and ferroptosis (13). Emerging evidence has demonstrated that dysregulated circRNAs exert a crucial role in human diseases, including cancer (14, 15), cardiovascular disease (16), diabetes (17), and Alzheimer’s disease (18). In fact, circRNAs are expressed in a specific manner in tissues and cells, indicating that they have distinct biological functions in various pathophysiological processes (7, 19, 20).

Wang et al. revealed the abnormal expression of circRNAs in HPV-related cervical cancer through RNA sequencing (RNA-seq) (21). Their data showed that 99 circRNAs are differentially expressed between cervical cancer tissues and adjacent non-tumor tissues. Ma et al. reported that 512 circRNAs are dysregulated in cervical cancer tissues (22). They further demonstrated that has_circ_000284 inhibition suppresses the growth and migration of cervical cancer cells *via* sponging miR-506 and de-repressing Snail-2 expression (22). Most circRNAs are located in cytoplasm (23, 24), and frequently act as competing endogenous RNAs (ceRNAs) by sponging miRNAs, and thereby increasing downstream gene expression. Hsa_circ_0021087, also known as circLMO1, is a newly identified circRNA in gastric cancer (25–27). However, the role of circLMO1 in gastric cancer is controversial. Yu et al. demonstrated that circLMO1 overexpression decreases gastric cancer cell proliferation and invasion (27). In contrast, Han et al. showed that circLMO1 promotes gastric cancer cell proliferation (28). The functional identification and characterization of circLMO1 in different types of tumors is necessary.

Ferroptosis is an iron-dependent programmed cell death (29, 30). More and more studies have shown that cancer cells are prone to ferroptosis (31). Sorafenib is an agonist of ferroptosis and the first-line therapy for advanced hepatocellular carcinoma (HCC) (32). Impressively, deferoxamine can markedly decrease the toxic effect of sorafenib on HCC cells (32). Jiang et al. demonstrated that oeanolic acid inhibits the proliferation of cervical cancer cells by promoting ACSL4-dependent ferroptosis (33). Up-regulated circRNA circEPSTI1 promotes cervical cancer growth by negatively regulating SLC7A11-dependent ferroptosis (13). There is increasing evidence that ferroptosis may be a potential target for cancer treatment.

In the study, the role of circLMO1 in cervical cancer progression and its relationship with ferroptosis was investigated. We demonstrated that circLMO1 expression was down-regulated in cervical cancer. CircLMO1 overexpression repressed cervical cancer growth and metastasis through sponging miR-4291, de-repressing ACSL4 expression, and thus accelerating ferroptosis.

## Materials and Methods

### Clinical Samples

Thirty-one cervical cancer tissues and matched normal tissues were obtained from the First Affiliated Hospital of Wenzhou Medical University with informed consent. The donor information is showed in **Table 1**. The protocol was conducted with the approval of the Hospital’s Protection of Human Subjects Committee.

**Table 1:** The correlation of circLMO1 level with clinicopathological characteristics in cervical cancer.

Characteristics	numbers	circLMO1 level			*p value*
		Low	High		
**Total cases**	31	16	15		
**Age**					0.593
≤55	12	7	5		
>55	19	10	9		
**Tumor size (cm)**					0.207
≤4.0	20	12	8		
>4.0	11	6	5		
**Histology**					0.319
Squamous	21	12	9		
Adenocarcinoma	10	4	6		
**FIGO stage**					0.027
Ib-IIa	13	5	8		
IIb-IIIa	18	11	7		
**Lymph node metastasis**					0.095
Yes	19	8	11		
No	12	5	7		

χ2 tests for all the analyses.

### Cell Culture

SiHa and Hela cell lines were purchased from China Center for Type Culture Collection (Wuhan, China). Normal human cervical epithelial cells (HUCEC), CaSki, and C33A cell lines were purchased from American Type Culture Collection (ATCC, VA, USA). All cells were in DMEM (Gibco, CA, USA) containing 10% FBS (Gibco) and 1× penicillin/streptomycin (Beyotime, Shanghai, China) at 37°C and 5% CO_2_ atmosphere.

### Quantitative Real-Time PCR (qPCR)

Total RNAs were collected using RNAsimple Total RNA Kit (DP419; Tiangen Biotech, Beijing, China) according to the manufacturer’s instructions, and quantified using a Nanodrop 1000 spectrophotometer (ThermoFisher Scientific, MA, USA). To determine the form of *circLMO1*, 4 μg of total RNA was digested with 8 U of RNase R (ThermoFisher Scientific) in 20 μL reaction buffer at 37°C for 0.5 h. Subsequently, the digested samples and mock samples were used as templates to verify the RNase R resistance by qPCR assay. First-stand cDNA was synthesized with SuperScript IV Reverse Transcriptase (18090010; Invitrogen, CA, USA) in the presence of Random 6 mers or Oligo dT Primer. qPCR was carried out in triplicate using the designated cDNA template and FastFire qPCR PreMix reagent (FP207; Tiangen Biotech) on Thermal Cycler Dice™ Real Time System III (TP950; TaKaRa). Thermocycling conditions were as followed: (i) 95°C for 60 s, (ii) 95°C 10 s, (iii) 60°C 15 s. Steps (ii) and (iii) were repeated for a total of 30 cycles. The 2^-ΔΔCt^ method was applied to calculate the expression level of each group and β-actin was used as an internal control. The sequences of specific primers were listed in **Supplementary Table S1**.

### Overexpression and RNA Interference (RNAi)

A recombinant lentivirus (Lv-circLMO1) encoding Exon 2 and Exon 3 of the *LMO1* gene, and its flanking intron contains reverse complement matching was obtained from Genechem (Shanghai, China) to overexpress circLMO1. The full length cDNA encoding DExH-Box Helicase 9 (DHX9) was inserted into pcDNA3.1 vector to overexpress DHX9 in cervical cancer cells. MiR-4291 mimics, siRNAs against circLMO1, DHX9, Acsl4, and miR-4291 were obtained from GenePharma (Shanghai, China). The sequences of siRNA and the miRNA mimics were showed in **Supplementary Table S1**.

CaSki cells and C33A cells in the logarithmic growth phase were plated into cell culture dish until cells reached approximately 70% confluence. Recombinant plasmids were transfected with Lipofectamine™ 3000 reagent (L3000015; Invitrogen, CA, USA), siRNAs and miRNA mimics were transfected with Lipofectamine™ RNAiMAX Reagent (13778030; Invitrogen). After transfection, cells were cultured at 37°C for 48h, followed by subsequent experiments.

### Cell Proliferation Assay

A commercial CCK-8 solution (GK10001; Glpbio, CA, USA) was used to measure cell viability and proliferation. For investigating the role of circLMO1 in regulating cell proliferation, CaSki cells (2×10^3^ cells/well) were seeded into a 96-well plate and overexpressed with circLMO1. At the indicated time points (0 h, 24 h, 48 h, and 72 h), cells were treated with 10 μL of CCK-8 reagent for 2 h, and then cell proliferation was assessed by measuring the absorbance at 450 nm on a microplate reader (Molecular Device, Sunnyvale, CA, USA). Moreover, C33A cells (2×10^3^ cells/well) were seeded into a 96-well plate and circLMO1 was knocked down. At the indicated time points, cells were treated with 10 μL of CCK-8 reagent for 2 h and cell proliferation was assessed. For assessing cell viability, CaSki cells were treated with Erastin (1 μM), Ferrostatin-1 (2 μM), ZVAD-FMK (10 μM), necrostatin-1 (12 μM), or disulfiram (6 μM) for 48 h after circLMO1 overexpression. Then cells were treated with 10 μL of CCK-8 reagent for 2 h and cell viability was calculated by measuring the absorbance at 450 nm on a microplate reader. Cell death inhibitors/agonists used in the study were purchased from Sigma-Aldrich (MO, USA).

### Colony Formation Assay

The circLMO1-overexpressed CaSki cells or circLMO1-knockdowned C33A cells were disaggregated into single cell suspensions. Live cells were counted by trypan blue staining, and were seeded (200 cells per well) into six-well plates. Then, cells were cultured in a complete medium containing 0.6% methylcellulose at 37°C for 14 days. After that, cells were washed with PBS for 3 times and fixed in 4% paraformaldehyde (PFA; Beyotime) for 3 min. Fixed colonies were stained with 0.5% crystal violet for 10 min.

### Transwell Invasion Assay

CaSki and C33A cells (2.0×10^4^ cells/200 μL serum-free DMEM medium) were treated with Lv-circLMO1 and siRNA-circLMO1, respectively, and then added in the Matrigel-coated upper chamber of Transwell chamber (24-well). DMEM medium (700 μL) supplemented with 10% FBS were added into the lower chamber. After 36 h of incubation, the non-invasive cells were wiped with a cotton swab and the cells on the lower surface were washed 3 times with PBS. After fixation with 4% PFA for 5 min, the cells were dyed with 0.5% crystal violet for 10 min. The cells on the lower surface were photographed (Nikon, Tokyo, Japan) under a microscope at 10 × magnification and counted in 5 independent fields with Fiji software (NIH, Bethesda, MD).

### Tumor-Bearing Mouse Model

Male BALB/c nude mice (6 weeks old) were purchased from Beijing Vital River Laboratory Animal Technology Co., Ltd. (Beijing, China). The mice were kept in a constant temperature (25°C) and pathogen-free room with free access to food and water *ad libitum*. The animal experiments were approved by the Ethics Committee for Animal Experimentation of The Second Affiliated Hospital and Yuying Children’s Hospital. Mice were euthanised with isoflurane inhalation. CaSki cells overexpressing circLMO1 (7×10^6^ cells/100 μL PBS) were injected subcutaneously into the flank of mice. Tumor growth was measured with a caliper 3 times a week and tumor-bearing mice were euthanised at 5 weeks after inoculation. Tumor volume (mm^3^) was estimated using the following formula: Tumor volume = ½ (longest diameter × shortest diameter^2^).

### *In Vivo* Metastasis Assay

CaSki cells stably overexpressed circLMO1 (3×10^6^ cells/200 μL PBS) were injected into nude mice through the tail vein. Ten weeks after the injection, a live animal fluorescence imaging system (Shanghai Unitech Instruments Co., Ltd., Shanghai, China) was used to observe lung metastases. Twenty minutes prior to image, mice were intraperitoneally injected with D-luciferin (1.5 mg/10 gm of mice) to provide as a luciferase substrate. Mice were euthanised and their lungs were fixed in 4% formaldehyde. The lung tissue was embedded in paraffin and cut into 6 μm sections for hematoxylin and eosin staining.

### Fluorescence *In Situ* Hybridization (FISH)

The subcellular localization of circLMO1 was evaluated using FISH assay. Specific RNA probes against cricMLO1 and miR-4291 was synthesized using FISH Tag™ RNA Kit (F32952; Invitrogen). CaSki and C33A cells (about 1.5×10^4^/well) were mounted on a coverslip and fixed with 4% PFA (Beyotime) at room temperature for 15 min. The cells were digested with protein K at 37°C for 1 h in the presence of glycine and acetic anhydride. Then, cells were treated with pre-hybridization solution for 90 min and treated with the probe (300 μL, 250 ng/mL) against cricMLO1 at 42°C overnight. Finally, cells were stained with DAPI for 5 min at room temperature before sealing. A fluorescence microscope (Keyence, Osaka, Japan) was used to capture the signals of cricMLO1, miR-4291 and the nucleus.

### RNA Immunoprecipitation (RIP)

EZ-Magna RIP kit (Millipore, MA, USA) was used to detect the binding of reverse complementary sequence in intron 1 (I1RC)/reverse complementary sequence in intron 3 (I3RC) to DHX9 and circLMO1 to AGO2. In brief, the magnetic beads were coated with rabbit anti-DHX9 antibody (A300-855A-M; Bethyl Laboratories, Inc., TX, USA) or rat anti-AGO2 antibody (SAB4200085; Sigma-Aldrich, MO) in RIP wash buffer at room temperature for 40 min. CaSki and C33A cells (8×10^6^) were lysed in RIPA lysis buffer (Beyotime) and the total protein was quantified by BCA method. Subsequently, the lysates were incubated with magnetic beads coated with either AGO2 antibody or DHX9 antibody in RIP buffer at 4°C overnight. The immunoprecipitated RNAs were isolated using protease K, and the enrichment of I1RC, I3RC, and circLMO1 was quantified by qPCR.

### Dual Luciferase Reporter Gene Detection

The wild-type predicted binding site or mutant binding site of circMLO1 with miR-4291 were cloned into the pGL3 vector (Vector Builder, Guangzhou, China) to construct pGL3-circLMO1-wt or pGL3-circLMO1-mut reporter plasmid. pGL3-ACSL4-3’UTR-wt and pGL3-ACSL4-3’UTR-mut recombinant plasmids were prepared by cloning the ACSL4-3’UTR or its mutant into the pGL3 vector. Before transfection, CaSki cells and C33A cells were seeded into 96-well plates and cultured for 24 h. Lipofectamine 3000 (Invitrogen) was used to transfect the designated plasmid with miR-4291 mimic or its mutant (miR-4291-mut) into cells, and the cells were cultured for another 48 h. Luciferase activities were quantified using Dual Luciferase Reporter Gene Assay Kit (RG027, Beyotime) according to the manufacturer’s instruction. Transfection efficiency was normalized by Renilla luciferase activity.

### RNA Pull-Down Assay

An RNA pull-down assay was performed to evaluate the direct combination between circLMO1 and miR-4291. Streptavidin magnetic beads (M2420; Solar Bio, Beijing, China) were labeled with biotinylated-miR-4291 (Sangon Biotech, Shanghai, China) at 4°C for 12 h. Approximately 0.5×10^7^ CaSki or C33A cells were lysed with commercial lysis buffer (P0013; Beyotime) supplemented with 40 U/mL RNasin (Tiangen Biotech). The lysate was reacted with RNA probe-labeled streptavidin magnetic beads for 3 h at room temperature. Finally, the circLMO1 content of the eluted complex was determined by qPCR.

### Iron (Fe^2+^), GSH, ROS, and MDA Assay

In order to determine the role of circLMO1 and miR-4291 in ferroptosis, the colorimetric determination kit (E-BC-K304-S; Elabscience, Hubei, China) for ferrous ion, GSH ELISA kit (ml077287; Shanghai Enzyme-Linked Biotechnology, Shanghai, China), lipid peroxidation MDA detection kit (S0131S; Beyotime) and C11-BODIPY (D3861; Invitrogen) were used to detect the levels of Fe^2+^, GSH, MDA, and ROS, respectively.

### Western Blot

The treated CaSki and C33A cells were lysed using RIPA Buffer (Beyotime), and the total protein concentration was measured by the BCA method using the BCA kit (BCA1-1KT, Sigma-Aldrich). About 20 μg of protein in each sample was electrophoresed on the 10% SDS-PAGE and then transferred to a PVDF membrane (0.45 μm, Millipore). The membrane was blocked in blocking solution (5% non-fat milk and 0.01% NaN_3_ in TBST) at room temperature for 90 min, and then probed with rabbit anti-LMO1 antibody (1:3000, ab137599, Abcam, MA, USA), rabbit anti-PTGS2 antibody (1:2000, SAB570072, Sigma-Aldrich), rabbit anti-ACSL4 antibody (1:5000, A305-358A, Bethyl Laboratories, TX, USA) and rabbit anti-β-actin antibody (1:2000, MA5-32479, Invitrogen) at 4°C overnight. After rinsing 3 times in TBST for 3 times, the membrane was labeled with HRP-conjured goat anti-rabbit IgG secondary antibody (1:5000, HA1001, HUA BIO, Shanghai, China) for 120 min at room temperature. The target protein was visualized using the ultra-high sensitivity ECL Kit (GK10008, Glpbio, CA, USA) and quantified by Fiji software. β-actin was used as the control.

### Statistical Analysis

Data are expressed as mean ± SEM from three independent experiments. All statistical analyses were implemented with GraphPad Prism 6.0 (GraphPad Software, CA, USA). Difference between groups were compared through one-way analysis of variance (ANOVA) followed by the Scheffé test, student’s t-test, and Fisher’s exact test. P-value less than 0.05 was statistically significant.

## Results

### Characterization of circLMO1 in Cervical Cancer

**Figure 1:**
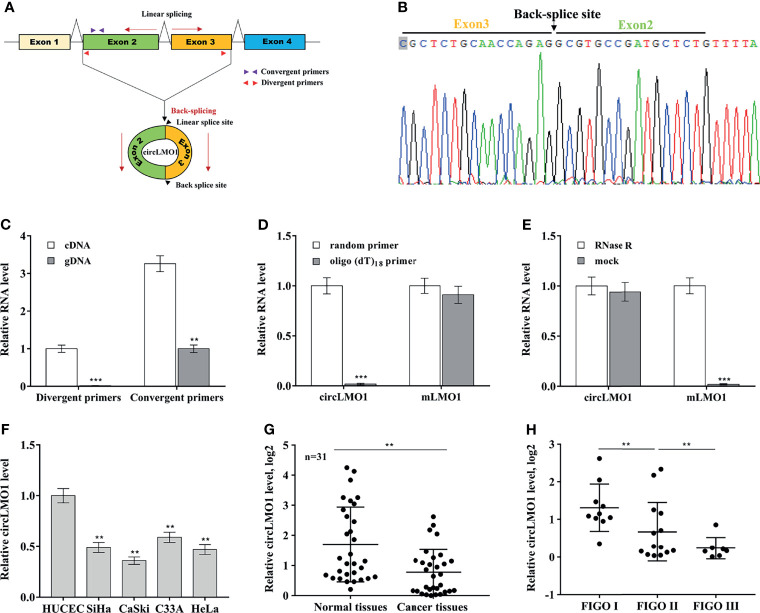
Features of circLMO1. **(A)** Schematic diagram of the formation of circLMO1. The qPCR primers used to evaluate the linear sequence of circLMO1, the convergent primers, are represented by red triangles. The qPCR primers used to evaluate the reverse splice site of circLMO1, the divergent primers, are represented by purple triangles. **(B)** Sanger sequencing of the reverse splice site of circLMO1. **(C)** qPCR analysis of circLMO1 level in DNA and cDNA derived from C33A cells using convergent primers and divergent primers. **(D)** Oligo (dT)18 primers or random primers was used to synthesize first-strand cDNA, and then qPCR analysis was performed to evaluate the level of circLMO1 and mLMO1 in these cDNAs. The value of the random primer is used as a reference. **(E)** Total RNA was treated with RNase R, and then qPCR analysis were performed to evaluate circLMO1 and mLMO1 level. **(F)** qPCR analysis was performed to assess circLMO1 expression in various cervical cancer cell lines (SiHa, CaSki, C33A, and HeLa) and normal cervical epithelial cell lines (HUCEC). **(G)** qPCR analysis was performed to assess circLMO1 level in cervical cancer tissues (n = 31) and matched normal tissues. **(H)** qPCR analysis was performed to assess circLMO1 level in different FIGO stages of cervical cancer. *p < 0.05, **p < 0.01, ***p < 0.001.

The biological role of circLMO1 in gastric cancer has been studied in previous studies (26, 27). However, the mechanism by which circLMO1 regulates cancer progression remains poorly understood. Here we evaluated the expression pattern, biological function and potential mechanism of circLMO1 in cervical cancer. Hsa_circ_0021087 is derived from the circularization of exon 2 and exon 3 of the *LMO1* gene, so it was named circLMO1 (**Figure 1A**). We used Sanger sequencing to verify the reverse splicing site in the PCR product of circLMO1 (**Figure 1B**). The results from qPCR analysis showed that although linear splicing sites existed in both complementary DNA (cDNA) and genomic DNA (gDNA), the reverse splicing site of circLMO1 only existed in cDNA (**Figure 1C**). To identify whether circLMO1 has poly (A) tail, reverse transcription PCR (RT-PCR) was performed using random primers or oligo (dT)18 primers. The results from qPCR showed that the level of circLMO1 in RT-PCR product of the oligo (dT)_18_ primer was lower than that of the random primer (**Figure 1D**), indicating that circLMO1 did not contain poly (A) tails. Furthermore, circLMO1 was RNase R-resistant, indicating that circLMO1 was a circular transcript, because linear transcripts were sensitive to RNase R (**Figure 1E**). To assess the relationship between circLMO1 expression and pathological features in cervical cancer, qPCR was used to assess circLMO1 level in cervical cancer tissues and cell lines. **Figure 1F** showed that circLMO1 expression in cervical cancer cell lines (SiHa, CaSki, C33A, and HeLa) was markedly down-regulated compared with normal cervical epithelial cells (HUCEC). CircLMO1 was also significantly down-regulated in tumor tissues (**Figure 1G**). Moreover, lower levels of circLMO1 were correlated with an increase in the International Federation of Gynecology and Obstetrics (FIGO) stage (**Figure 1H**).

### Intron Pairing Drives the Circularization of circLMO1

**Figure 2:**
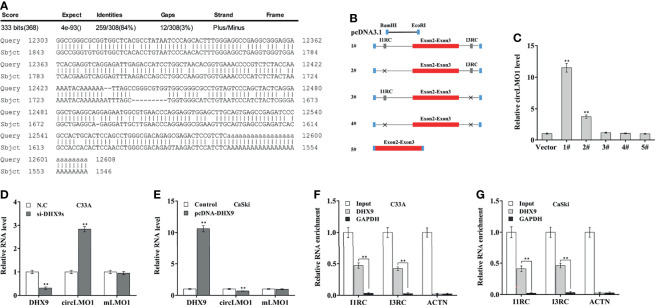
Intron pairing drives the cyclization of circLMO1. **(A)** Alignment of I1RC and I3RC located in the flanking intron of circLMO1. **(B)** Schematic diagram of the recombinant plasmid used to explore the circularization of circLMO1. The genomic region (red bar) of circLMO1 and its wildtype flanking introns (black line) containing I1RC and I3RC (grey bars) were cloned into the pcDNA vector (1#). A black cross indicates a corresponding absence. **(C)** qPCR analysis was performed to assess circLMO1 level in CaSki cells treated with different recombinant plasmids (1# to 5#). qPCR analysis of circLMO1 and mLMO1 levels in C33A cells after DHX9 knockdown **(D)** and CaSki cells after DHX9 overexpression **(E)**. DHX9 antibody was used for RIP assay to evaluate the direct combination of DHX9 with I1RC and I3RC in C33A **(F)** and CaSki cells **(G)**. **p < 0.01.

Most circRNAs are derived from the circularization of exon with flanking introns, which commonly contain reverse complementary matches (RCMs) (34, 35). RCMs can form base-pairing and hairpins between flanking introns to promote back-splicing. By aligning the sequences of intron 1 and intron 3 with the Basic Local Alignment Search Tool (BLAST), the high RCMs were identified (**Figure 2A**). To investigate whether the circularization of circLMO1 was facilitated by I1RC (reverse complementary sequence in intron 1) and I3RC (reverse complementary sequence in intron 3), the 5 sequences were separately cloned into the pcDNA3.1 vector (**Figure 2B**): 1#, Exon 2 and 3 with wild type of I1RC and I3RC; 2#, Exon 2 and 3 with I3RC (without I1RC); 3#, Exon 2 and 3 with I1RC (without I3RC); 4#, Exon 2 and 3 with neither I1RC nor I3RC; 5#, Exon 2 and 3 with no flanking introns. After transfection of these recombinant plasmids, the results from qPCR analysis showed that the wild type plasmid (1#), but not the 3#, 4# and 5# plasmids, could overexpress circLMO1 (**Figure 2C**). Besides, 2# plasmid partly overexpressed circLMO1. This may be because intron 1 is a long flanking intron (37.922 kb) and thus contains multiple RCMs except I1RC (**Supplementary Figure S1**). These results indicate that I3RC is indispensable for the circularization of circLMO1.

DExH-Box Helicase 9 (DHX9), quaking (QKI), and adenosine deaminase 1 acting on RNA (ADAR1) are RNA-binding proteins (RBPs) that were demonstrated to extensively control circRNA biogenesis (36, 37). Data from the GEPIA database (http://gepia.cancer-pku.cn/) showed that DHX9 level was increased in cervical cancer tissues compared with normal control, ADAR1 level was unchanged, and QKI level was decreased in tumor tissues (**Supplementary Figures S2A–C**). Knockdown of DHX9, but not ADAR1 and QKI, significantly up-regulated circLMO1 expression in C33A cells (**Figure 2D**, **Supplementary Figure S2D**). In contrast, overexpression of DHX9, but not ADAR1 and QKI, down-regulated circLMO1 expression in CaSki cells (**Figure 2E**, **Supplementary Figure S2E**). Furthermore, RIP assay using a DHX9 antibody exhibited a significant enrichment of I1RC and I3RC in C33A and CaSki cells (**Figures 2F, G**). These data indicate that intron pairing drives the circularization of circLMO1, and up-regulated DHX9 leads to a significant decrease in circLMO1 in cervical cancer cells.

### CircLMO1 Inhibits Cervical Cancer Growth and Metastasis

**Figure 3:**
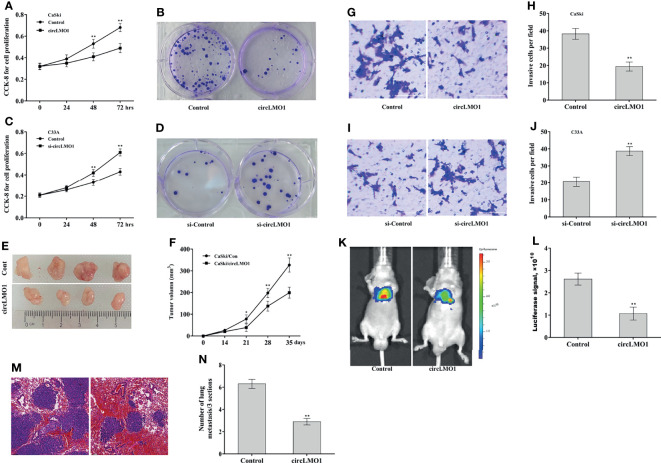
CircLMO1 inhibits cervical cancer growth and metastasis. CCK-8 **(A)** and colony formation assay **(B)** were carried out to assess CaSki cell proliferation and growth after circLMO1 overexpression. CCK-8 **(C)** and colony formation assay **(D)** were carried out to assess C33A cell proliferation and growth after circLMO1 knockdown. **(E, F)** CaSki cells stably overexpressed with circLMO1 were injected subcutaneously into nude mice, and tumor volume was calculated at different time points (n = 4). Transwell invasion assay of CaSki cells **(G, H)** after circLMO1 overexpression and C33A cells **(I, J)** after circLMO1 knockdown. Scale bar = 100 µm. **(K, L)** CaSki cells stably overexpressed circLMO1 were injected into nude mice through tail vein, and *in vivo* bioluminescence imaging was used to assess cancer metastasis at 10 weeks (n = 4). **(M)** H&E staining in lung tissue was used to assess lung metastasis. Scale bar = 100 µm. **(N)** The number of lung metastatic nodules at 10 weeks. *p < 0.05, **p < 0.01.

The level of endogenous circLMO1 was the highest in C33A cells and lowest in CaSki cells (**Figure 1F**). To investigate the biological role of circLMO1, circLMO1 was overexpressed in CaSki cells and knocked down in C33A cells, and then cell proliferation and invasion were assessed both *in vitro* and *in vivo*. The ectopic expression and depletion of circLMO1 were verified by qPCR (**Supplementary Figures S3A, B**). The results from CCK-8 (**Figure 3A**) and colony formation assay (**Figure 3B**) showed that forced expression of circLMO1 significantly decreased CaSki cell proliferation and growth. In contrast, circLMO1 knockdown accelerated C33A cell proliferation and growth (**Figures 3C, D**). We next explored the role of circLMO1 in tumor growth *in vivo*. As shown in **Figures 3E, F**, circLMO1 overexpression in CaSki cells significantly suppressed tumor growth of cervical cancer xenografts in nude mice. The role of circLMO1 in regulating cell invasion *in vitro* and tumor metastasis *in vivo* were further investigated. Forced expression of circLMO1 inhibited CaSki cell invasion (**Figures 3G, H**), while circLMO1 depletion promoted C33A cell invasion (**Figures 3I, J**). The CaSki cells stably overexpressed circLMO1 were injected into nude mice through tail vein to establish a cervical cancer lung metastasis model. **Figures 3K, L** showed that circLMO1 overexpression significantly repressed cancer metastasis *in vivo* compared with control. Histological analysis further showed that circLMO1-overexpressed CaSki cells formed less and smaller lung metastatic nodules compared with control (**Figures 3M, N**).

### CircLMO1 Promotes Cervical Cancer Cell Ferroptosis

**Figure 4:**
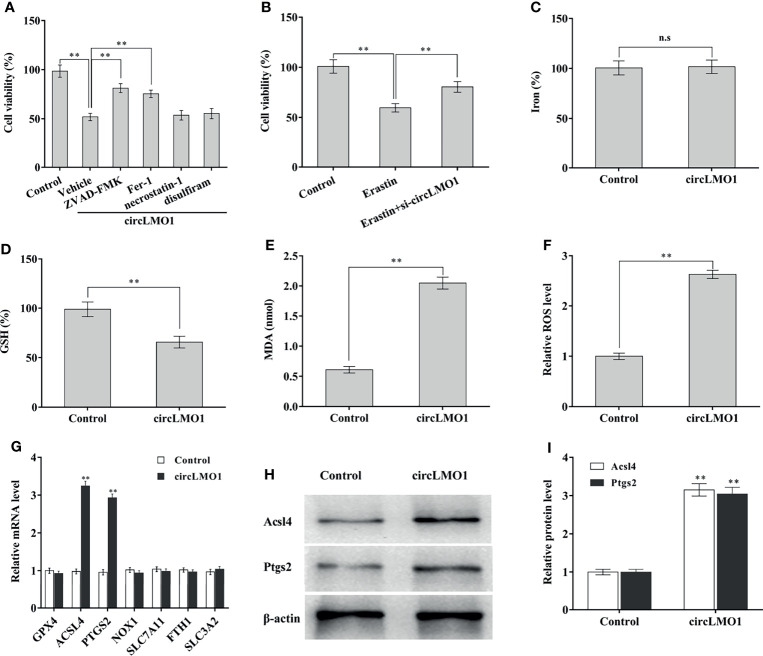
CircLMO1 promotes cervical cancer cell ferroptosis. **(A)** CCK-8 assay was carried out to assess CaSki cell viability after circLMO1 overexpression in the presence or absence of ZVD-FEK, Fer-1, Necrostatin-1 and Disulfiram. **(B)** CCK-8 assay was carried out to assess CaSki cell viability after Erastin treatment in the presence or absence of circLMO1 overexpression. Relative iron concentration **(C)**, GSH content **(D)**, MDA content **(E)**, and ROS level **(F)** was evaluated in CaSki cells after circLMO1 overexpression. **(G)** qPCR analysis was performed to assess the expression of ferroptosis-related genes in CaSki cells after circLMO1 overexpression. **(H, I)** Western blot and quantitative analysis of ACSL14 and PTGS2 protein levels in CaSki cells after circLMO1 overexpression. *p < 0.05, **p < 0.01, n.s, not significant.

CircLMO1-induced cell death pattern was next explored. To this end, circLMO1 was overexpressed in CaSki cells in the presence of Ferrostatin-1 (Fer-1, a ferroptosis inhibitor), ZVAD-FMK (an apoptosis inhibitor), necrostatin-1 (a necroptosis inhibitor), or disulfiram (a pyroptosis inhibitor), and then cell viability was assayed. **Figure 4A** showed that circLMO1-mediated cell death was significantly repressed by Fer-1 or ZVAD-FMK, but not necrostatin-1 and disulfiram, indicating that circLMO1 promoted cervical cancer cell death by triggering ferroptosis and apoptosis. As a novel type of cell death related to cancer, ferroptosis is closely associated with cervical cancer progression (13, 33, 38). Here we focused on the effect of circLMO1 on ferroptosis. **Figure 4B** showed that Erastin (an activator of ferroptosis) reduced C33A cell viability, while circLMO1 inhibition prevented this effect, indicating that ferroptosis is an important pattern of cervical cancer cell death, and that circLMO1 accelerated cervical cancer cell death by regulating ferroptosis. To define the role of circLMO1 in ferroptosis, iron concentration, GSH and MDA content, and ROS level were assayed after circLMO1 overexpression. **Figures 4C**–**F** showed that circLMO1 was not associated with iron concentration in CaSki cells, but circLMO1 decreased GSH content, and increased MDA content and ROS level.

To reveal the mechanism by which circLMO1 triggers ferroptosis, the expression of ferroptosis-related mRNAs was assayed in CaSki cells after circLMO1 overexpression. As shown in **Figure 4G**, circLMO1 overexpression resulted in a significant increase of ACSL4 and PTGS2 mRNA levels. Western blot analysis demonstrated that circLMO1 increased ACSL4 and PTGS2 protein levels (**Figures 4H, I**). These results suggest that circLMO1 facilitates cervical cancer cell ferroptosis at least in part by increasing ACSL4 or PTGS2 expression.

### CircLMO1 Acts as a Sponge for miR-4291 in Cervical Cancer Cells

**Figure 5:**
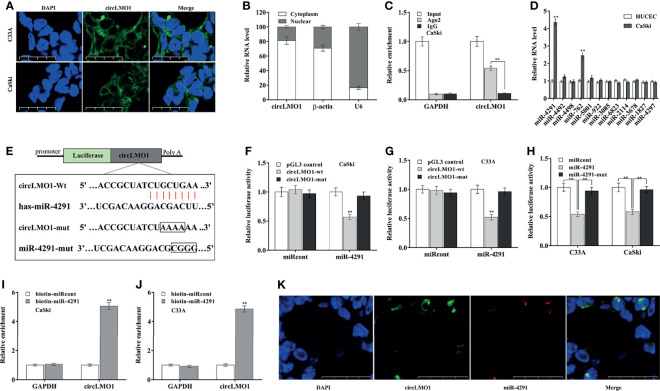
CircLMO1 acts as a sponge for miR-4291 in cervical cancer cells. **(A)** FISH assay were carried out to assess circLMO1 cell localization in C33A and CaSki cells. The RNA probe targeting circLMO1 was stained green and the nucleus was stained blue. **(B)** qPCR analysis of circLMO1 levels in the cytoplasm and nucleus of C33A cells. U6 was used as a positive control in the nucleus, and β-actin was used as a positive control in the cytoplasm. **(C)** AGO2 antibody was used to perform RIP assay to determine the combination of circLMO1 and AGO2 in CaSki cells. **(D)** qPCR analysis was performed to assess miRNAs expression in CaSki and HUCEC cells. **(E)** Schematic diagram of the predicted miR-4291-circLMO1 interaction. **(F–H)** After co-transfection with miR-4291 (or its mutant) and circLMO1-wt luciferase reporter gene (or its mutant), luciferase activity was assessed in CaSki and C33A cells. After transfection with biotin-labelled miR-4291, qPCR analysis was performed to assess circLMO1 level in the streptavidin precipitation complex from CaSki cells **(I)** or C33A cells **(J)**. **(K)** The co-localization of circLMO1 and miR-4291 analysed through double FISH assay in C33A cells. *p < 0.05, **p < 0.01.

The circRNAs located in the cytoplasm usually act as ceRNAs to regulate mRNA levels *via* sponging miRNAs (39). The subcellular localization of circLMO1 was examined by qPCR and FISH. **Figures 5A, B** showed that circLMO1 was mostly located in the cytoplasm. RIP assay using argonaute 2 (AGO2) antibody showed that circLMO1 was markedly enriched by AGO2 antibody (**Figure 5C**). These results indicated that circLMO1 may act as a ceRNA. The bioinformatics analysis was carried out using miRDB tool (http://mirdb.org/mirdb/index.html) to predict the potential miRNAs sponged by circLMO1, and 12 miRNAs were identified (**Supplementary Table S2**). Among them, miR-4291 and miR-762 were significantly increased in cervical cancer cells compared with normal cervical epithelial cells (**Figure 5D**). The miR-4291 was selected for further research because miR-4291 expression was higher than miR-762.

To validate the direct combination of circLMO1 with miR-4291 (**Figure 5E**), the recombinant plasmid of pGL3-circLMO1-wt or its mutant (pGL3-circLMO1-mut) was co-transfected with miR-4291 into cervical cancer cells. **Figures 5F, G** revealed that the luciferase activity of pGL3-circLMO1-wt was significantly repressed after miR-4291 transfection, but the luciferase activity of pGL3-circLMO1-mut was not affected by miR-4291 (**Supplementary Figure S3C**). Meanwhile, miR-4291 mutant lost the role in repressing luciferase activity of pGL3-circLMO1-wt in CaSki and C33A cells (**Figure 5H**). The direct combination of circLMO1 with miR-4291 was further assessed through RNA pull-down assay with biotin-labeled miR-4291. As shown in **Figures 5I, J**, a marked enrichment of circLMO1 was observed in pull-down assay with biotin-labelled miR-4291. Moreover, the co-localization of circLMO1 and miR-4291 was verified through dual FISH assay (**Figure 5K**). These results indicate that circLMO1 acts as a sponge for miR-4291 in cervical cancer cells.

### CircLMO1 Increases ACSL4 Expression by Sponging miR-4291

**Figure 6:**
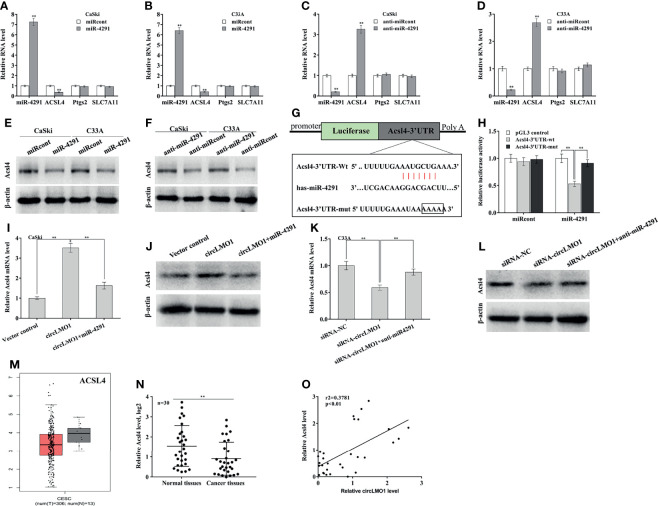
CircLMO1 increases ACSL4 expression by sponging miR-4291. qPCR analysis of Acsl4, Ptgs2, and Slc7a11 level in CaSki **(A)** and C33A cells **(B)** after miR-4291 overexpression. qPCR analysis of Acsl4, Ptgs2, and Slc7a11 level in CaSki **(C)** and C33A cells **(D)** after miR-4291 inhibition. **(E)** Western blot analysis of ACSL4 protein level in CaSki and C33A cells after miR-4291 overexpression. **(F)** Western blot analysis of ACSL4 protein level in CaSki and C33A cells after miR-4291 inhibition. **(G)** Schematic illustration of the predicted miR-4291-ACSL4 interactions. **(H)** Luciferase activities were assessed in C33A cells after co-transfection with miR-4291 and Acsl4-3’UTR-wt luciferase reporters (or its mutant). qPCR **(I)** and western blot analysis **(J)** of Acsl4 expression in CaSki cells after circLMO1 overexpression in the presence or absence of miR-4291 mimics. qPCR **(K)** and Western blot analysis **(L)** of Acsl4 expression in C33A cells after circLMO1 knockdown in the presence or absence of miR-4291 inhibitor. **(M)** Acsl4 levels in cervical squamous cell carcinoma and endocervical adenocarcinoma were analysed *via* GEPIA database. **(N)** qPCR analysis of Acsl4 level in cervical cancer tissues (n = 30) and matched normal tissues. **(O)** The positive association between the circLMO1 level and the miR-4291 level in 30 cervical cancer tissues. *p < 0.05, **p < 0.01.

TargetScan7.1 tool (http://www.targetscan.org/vert_71/) was applied to predict the target genes of miR-4291. There are 5084 genes possibly targeted by miR-4291. Among 5084 genes, ACSL4, PTGS2, and SLC7A11 are ferroptosis-related genes (**Supplementary Figure S4A**). The role of miR-4291 in regulating these genes expression was next assessed. miR-4291 overexpression significantly decreased ACSL4 expression in CaSki and C33A cells (**Figures 6A, B**), while miR-4291 inhibition increased ACSL4 expression (**Figures 6C, D**). The miR-4291 did not affect the expression of PTGS2 and SLC7A11 (**Figures 6A–D**, **Supplementary Figures S4B, C**). Western blot analysis also demonstrated that miR-4291 negatively regulated ACSL4 protein level in CaSki and C33A cells (**Figures 6E, F**), suggesting that ACSL4 may be a target gene of miR-4291. The recombinant plasmids of pGL3-ACSL4-3’UTR-wt or pGL3-ACSL4-3’UTR-mut was constructed by cloning ACSL4-3’UTR or its mutant into pGL3 (**Figure 6G**). **Figure 6H** showed that the luciferase activity of pGL3-ACSL4-3’UTR-wt was markedly decreased after miR-4291 transfection, but 4 nucleotides mutation in ACSL4-3’UTR resulted in complete loss of the repressive role.

Based on the above findings, we speculated that circLMO1 might increase ACSL4 expression in a miR-4291-dependent manner. As expected, circLMO1 enhanced the mRNA and protein level of ACSL4 in CaSki cells, while miR-4291 reversed the effect (**Figures 6I, J**). CircLMO1 depletion repressed ACSL4 expression, while miR-42391 inhibition significantly restored ACSL4 expression (**Figures 6K, L**). By analyzing the GEPIA database, it was found that ACSL4 level was down-regulated in cervical squamous cell carcinoma and cervical adenocarcinoma compared with normal tissues (**Figure 6M**). ACSL4 was also significantly down-regulated in tumor tissues compared with matched normal tissues (**Figure 6N**). Moreover, the ACSL4 level was positively associated with the circLMO1 level in 30 cervical cancer tissues (**Figure 6O**, r2 = 0.3781, p<0.01).

### CircLMO1 Regulates the Ferroptosis, Proliferation, and Invasion of Cervical Cancer Cells in a miR-4291/ACSL4-Dependent Manner

**Figure 7:**
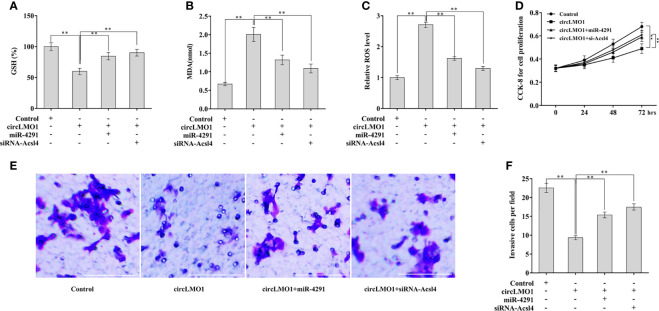
CircLMO1 promotes cervical cell ferroptosis, and inhibited cell proliferation and invasion in a miR-4291/ACSL4-dependent manner. Relative GSH content **(A)**, MDA content **(B)**, and ROS level **(C)** in CaSki cells were assessed after circLMO1 overexpression in the presence or absence of miR-4291 mimics (or siRNA-Acsl4). **(D)** CaSki cell proliferation were assessed using CCK-8 assay after circLMO1 overexpression in the presence or absence of miR-4291 mimics (or siRNA-Acsl4). **(E, F)** CaSki cell invasion were assessed using transwell invasion assay after circLMO1 overexpression in the presence or absence of miR-4291 mimics (or siRNA-Acsl4). **p < 0.01.

Finally, we assessed the role of circLMO1/miR-4291/ACSL4 axis in regulating cervical cancer cell ferroptosis, proliferation, and invasion. CircLMO1 decreased GSH content in CaSki cells, whereas miR-4291 overexpression or ACSL4 depletion restored GSH content (**Figure 7A**). Similarly, circLMO1 increased MDA content and ROS level in CaSki cells, whereas miR-4291 overexpression or ACSL4 depletion significantly reversed the effect (**Figures 7B, C**). More important, circLMO1 repressed CaSki cell proliferation and invasion, whereas miR-4291 overexpression or ACSL4 depletion significantly alleviated these effects (**Figures 7D–F**). Taken together, these data demonstrate that circLMO1 inhibits cervical cancer cell proliferation and invasion by facilitating miR-4291/ACSL4-mediated ferroptosis.

## Discussion

Due to its unique structure, circRNA has become a cancer treatment target. However, the role of circRNAs in the progression of cervical cancer remains unclear. The current study demonstrated that, i) circLMO1 expression was down-regulated in cervical cancer, ii) circLMO1 inhibited cervical cancer growth and metastasis, iii) circLMO1 promoted cervical cancer cell ferroptosis, iv) circLMO1 acted as a sponge for miR-4291 in cervical cancer cells, v) circLMO1 up-regulated *ACSL4* expression through sponging miR-4291, and vi) circLMO1 inhibited cervical cancer cell proliferation and invasion by promoting miR-4291/*ACSL4*-dependent ferroptosis. These findings revealed the key function of circLMO1/miR-4291/*ACSL4*/ferroptosis axis on cervical cancer, and therefore provided a potential opportunity for developing new drugs to treat such disease.

The data from transcriptome and bioinformatics analysis showed that a large number of circRNAs are expressed in tumor tissues, and dysregulated circRNAs play a key role in tumor progression. Li et al. reported that a total of 80,000 circRNAs are transcribed in cervical cancer tissues and adjacent normal tissues (40). Among these circRNAs, about 25,000 circRNAs are differentially expressed between tumor tissues and normal tissues (40). Ma et al. revealed that 512 circRNAs are differentially expressed between cervical cancer cells and normal cervical epithelial cells (22). CircRNA-000284 is increased in cervical cancer cells, which acts as a tumor activator by sponging miR-506 and de-inhibiting the expression of *Snail 2* (22). The dysregulation of non-coding RNAs (circRNA, lncRNA, and miRNA) induced by HPV E6 or E7 oncoprotein is a critical factor in its carcinogenic activity (41, 42). Zheng et al. found that 526 circRNAs are differentially expressed in Caski cells after E7 knockdown (42). E6/E7 oncoprotein increases DHX9 expression by inhibiting lncRNA-CCDST (43). In this study, we showed that DHX9 expression in cervical cancer tissues is up-regulated. DHX9 knockdown enhances circLMO1 expression in cervical cancer cells, while DHX9 overexpression down-regulates circLMO1 expression. RIP assay using DHX9 antibody showed that intron 1 and intron 3 of LMO1 are significantly enriched, indicating that up-regulated DHX9 results in a decrease in circLMO1 in cervical cancer.

CircLMO1 has been confirmed to play a carcinogenic role in gastric cancer. At present, the mechanism by which circLMO1 in regulating gastric cancer progression has not been fully elucidated. In addition, the influence of circLMO1 on gastric cancer is still controversial. Yu et al. showed that circLMO1 inhibits gastric cancer cell proliferation and invasion (27). In contrast, Han et al. demonstrated that circLMO1 promotes gastric cancer cell proliferation (28). In this study, we demonstrated that forced expression of circLMO1 inhibits the proliferation, colony formation, and invasion of cervical cancer cells both *in vitro* and *in vivo*, while circLMO1 knockdown accelerates cervical cancer cell proliferation and invasion.

Although multiple genes, such as *ACSL4, PTGS2, NOX1, GPX4, FTH1*, and *SLC7A11*, have been shown to be associated with ferroptosis (44), it is unclear how ferroptosis is genetically programmed in cancers. Wu et al. for the first time revealed the association between circRNA and ferroptosis in cervical cancer (13). They demonstrated that circEPSTI1 promotes cervical cancer cell proliferation by regulating SLC7A11-mediated ferroptosis. In this study, we found that circLMO1-mediated cell death is significantly repressed by Fer-1, indicating that circLMO1 promotes cervical cancer cell death by triggering ferroptosis. Different from above study, circLMO1 do not regulate SLC7A11 expression. CircLMO1 triggers ferroptosis through sponging miR-4291, thereby enhancing *ACSL4* expression in cervical cancer cells. As expected, miR-4291 overexpression or *ACSL4* knockdown effectively reverses the role of circLMO1 in promoting ferroptosis.

Conclusion: circLMO1 is downregulated in cervical cancer and circLMO1 overexpression inhibits cervical cancer growth and metastasis by promoting miR-4291/*ACSL4*-mediated ferroptosis.

## Data Availability Statement

The original contributions presented in the study are included in the article/**Supplementary Material**. Further inquiries can be directed to the corresponding authors.

## Ethics Statement

The studies involving human participants were reviewed and approved by Ethics Committee for Animal Experimentation of The Second Affiliated Hospital and Yuying Children’s Hospital. The patients/participants provided their written informed consent to participate in this study. The animal study was reviewed and approved by Ethics Committee for Animal Experimentation of The Second Affiliated Hospital and Yuying Children’s Hospital. Written informed consent was obtained from the individual(s) for the publication of any potentially identifiable images or data included in this article.

## Author Contributions

RO, JL, and R-sG contributed to conception and design of the study. YW, SL, and LW performed the statistical analysis. ML, TL, and YX wrote the first draft of the manuscript. RO and JL wrote sections of the manuscript. All authors contributed to manuscript revision, read, and approved the submitted version.

## Funding

This work was supported by grants from the National Natural Science Foundation of China (No. 81871129, 82072862, 82072863, 82002727, 82172883).

## Conflict of Interest

The authors declare that the research was conducted in the absence of any commercial or financial relationships that could be construed as a potential conflict of interest.

## Publisher’s Note

All claims expressed in this article are solely those of the authors and do not necessarily represent those of their affiliated organizations, or those of the publisher, the editors and the reviewers. Any product that may be evaluated in this article, or claim that may be made by its manufacturer, is not guaranteed or endorsed by the publisher.

## Supplementary Material

Supplementary Figure 1 The high RCMs were identified through alignment of the intron 1 and intron 3 sequences with BLAST.

Supplementary Figure 2 ADAR1 and QKI did not regulate circLMO1 expression. Data from the GEPIA database showed that DHX9 level **(A)** was increased in cervical cancer tissues, ADAR1 level was unchanged **(B)**, and QKI level **(C)** was decreased in tumor tissues compared with normal control. qPCR analysis of circLMO1 and mLMO1 levels in C33A cells after ADAR1 or QKI knockdown **(D)**, and in CaSki cells after ADAR1 or QKI overexpression **(E)**. **p < 0.01.

Supplementary Figure 3 qPCR analysis of circLMO1 and miR-4291. **(A)** CaSki cells were treated with Lv-circLMO1 or Lv-cont and then circLMO1 expression was assessed using qPCR. **(B)** C33A cells were treated with siRNA-circLMO1 or siRNA-control and then circLMO1 expression was assessed using qPCR. **(C)** C33A cells were treated with miR-4291 mimics or anti-miR-4291, and then miR-4291 expression was assessed using qPCR. **p< 0.01.

Supplementary Figure 4 The predicted target gene of miR-4291. **(A)** TargetScan7.1 tool was used to predict the target genes of miR-4291. There are 5084 genes possibly targeted by miR-4291. Among 5084 genes, 3 genes (ACSL4, PTGS2, and SLC7A11) are ferroptosis-related genes. **(B)** C33A and CaSki cells were treated with miR-4291 mimics and then the protein level of Slc7a11 and Ptgs2 was assessed using western blot. **(C)** C33A and CaSki cells were treated with anti-miR-4291 and then the protein level of Slc7a11 and Ptgs2 was assessed using western blot.
