# Supplementary figures and images for: Circular RNA circLMO1 Suppresses Cervical Cancer Growth and Metastasis by Triggering miR-4291/ACSL4-Mediated Ferroptosis
Source: Front Oncol. 2022 Mar 7;12:858598. doi: 10.3389/fonc.2022.858598 (PMC8936435; doi:10.3389/fonc.2022.858598)

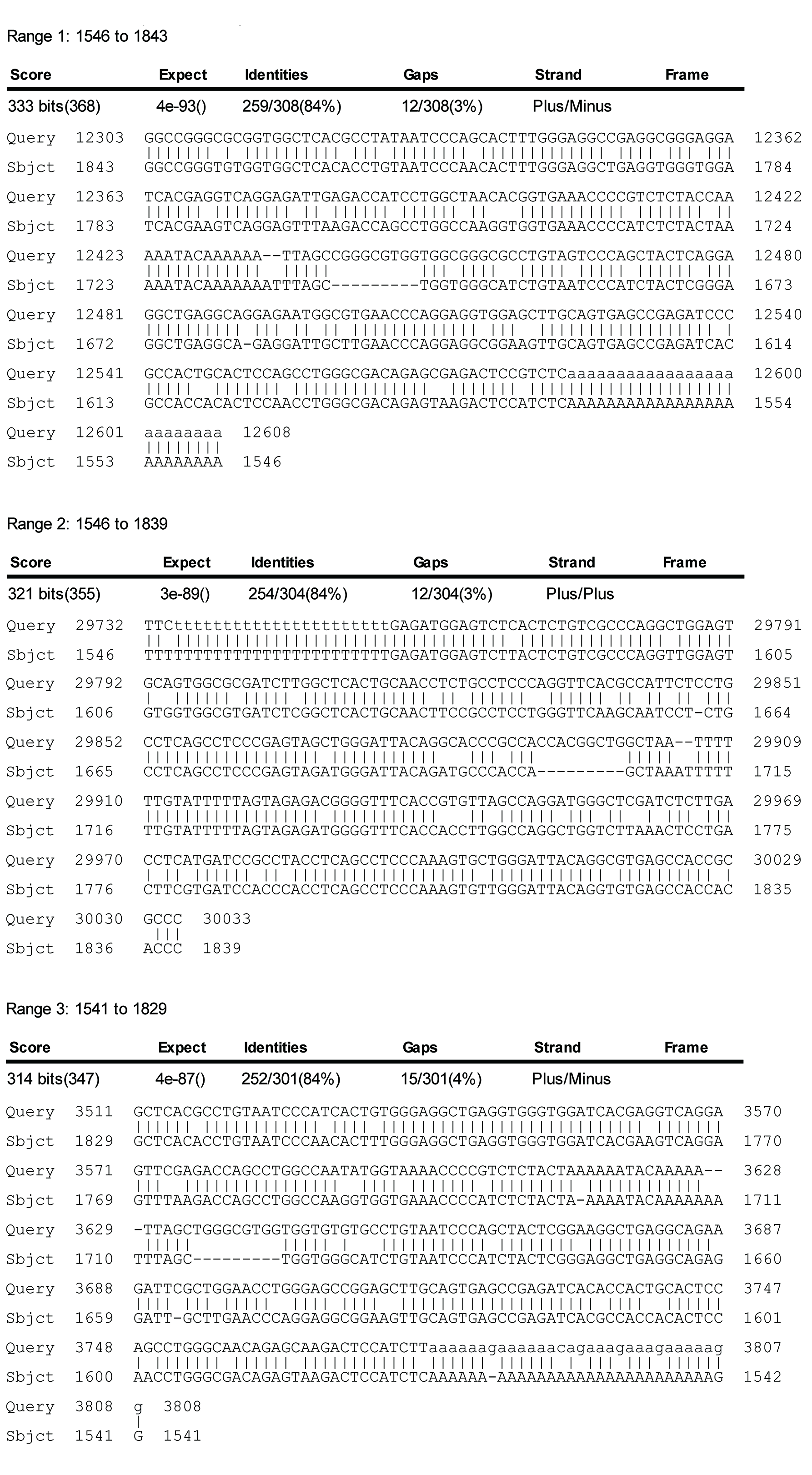

Supplement: Supplementary Figure 1 — The high RCMs were identified through alignment of the intron 1 and intron 3 sequences with BLAST. [file Image_1.tif]

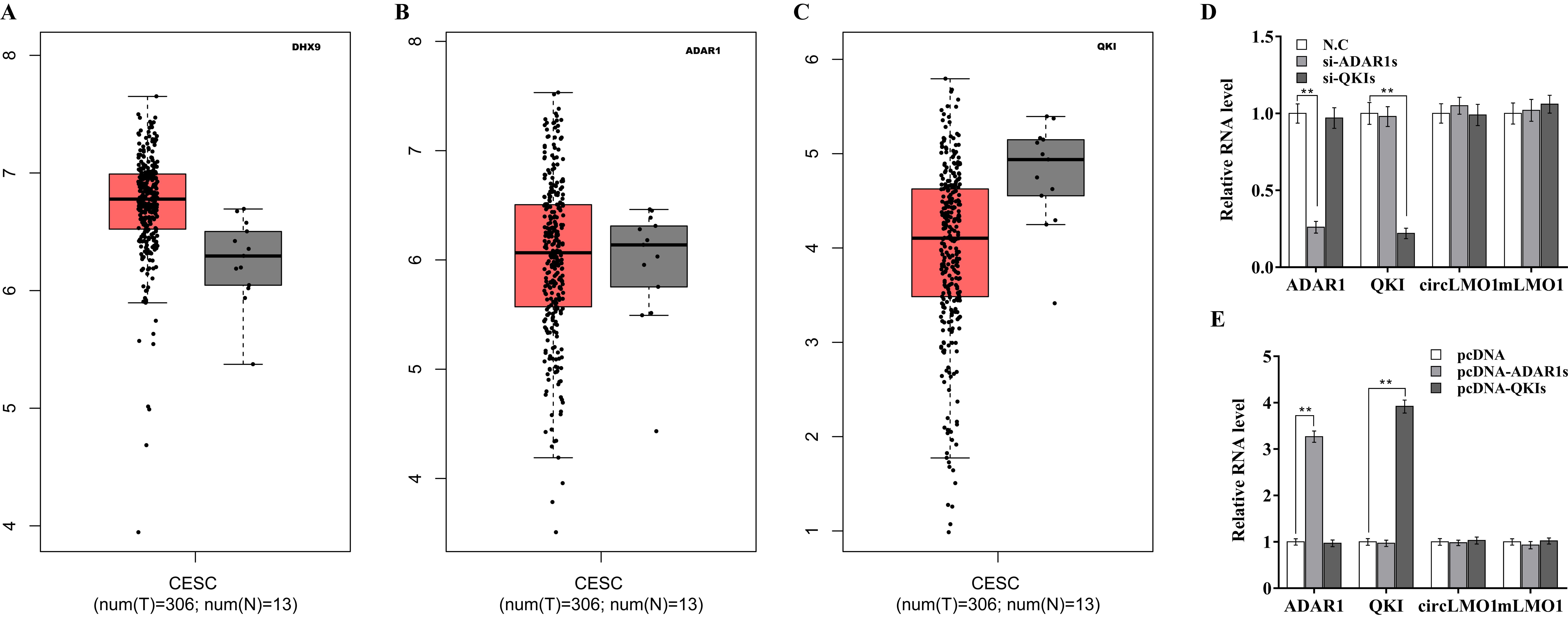

Supplement: Supplementary Figure 2 — ADAR1 and QKI did not regulate circLMO1 expression. Data from the GEPIA database showed that DHX9 level (A) was increased in cervical cancer tissues, ADAR1 level was unchanged (B), and QKI level (C) was decreased in tumor tissues compared with normal control. qPCR analysis of circLMO1 and mLMO1 levels in C33A cells after ADAR1 or QKI knockdown (D), and in CaSki cells after ADAR1 or QKI overexpression (E). **p < 0.01. [file Image_2.tif]

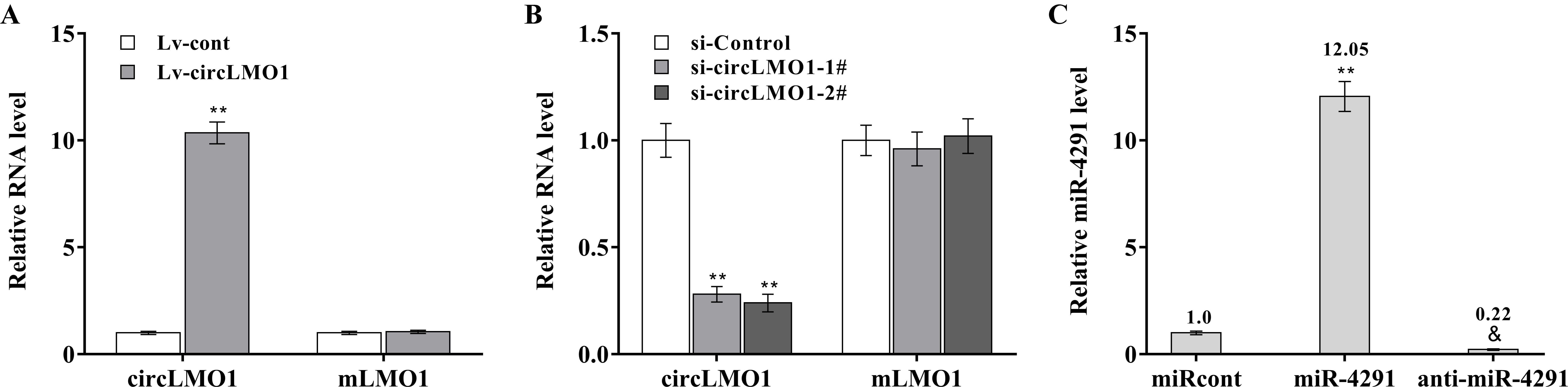

Supplement: Supplementary Figure 3 — qPCR analysis of circLMO1 and miR-4291. (A) CaSki cells were treated with Lv-circLMO1 or Lv-cont and then circLMO1 expression was assessed using qPCR. (B) C33A cells were treated with siRNA-circLMO1 or siRNA-control and then circLMO1 expression was assessed using qPCR. (C) C33A cells were treated with miR-4291 mimics or anti-miR-4291, and then miR-4291 expression was assessed using qPCR. **p< 0.01. [file Image_3.tif]

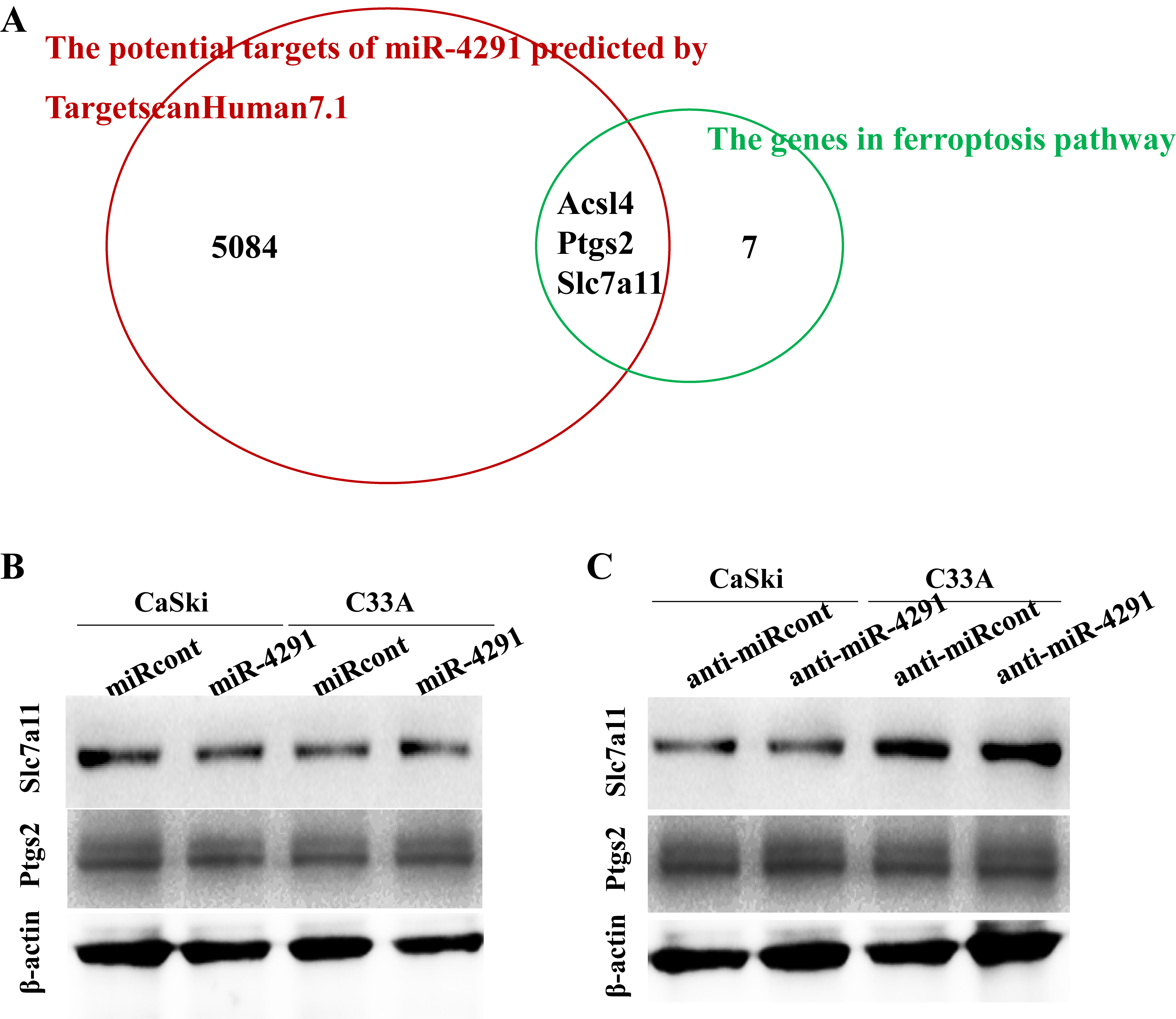

Supplement: Supplementary Figure 4 — The predicted target gene of miR-4291. (A) TargetScan7.1 tool was used to predict the target genes of miR-4291. There are 5084 genes possibly targeted by miR-4291. Among 5084 genes, 3 genes (ACSL4, PTGS2, and SLC7A11) are ferroptosis-related genes. (B) C33A and CaSki cells were treated with miR-4291 mimics and then the protein level of Slc7a11 and Ptgs2 was assessed using western blot. (C) C33A and CaSki cells were treated with anti-miR-4291 and then the protein level of Slc7a11 and Ptgs2 was assessed using western blot. [file Image_4.tif]
