# Supplementary material for: Circular RNA circLMO1 Suppresses Cervical Cancer Growth and Metastasis by Triggering miR-4291/ACSL4-Mediated Ferroptosis
Source: Front Oncol. 2022 Mar 7;12:858598. doi: 10.3389/fonc.2022.858598 (PMC8936435; doi:10.3389/fonc.2022.858598)
Supplement: Supplementary file 5 [file DataSheet_1.docx]

| Gene | Sense (5’-3’) | Antisense (5’-3’) | | |  | |
| --- | --- | --- | --- | --- | --- | --- |
| miR-4291 | UUCAGCAGGAACAGCU |  |  |  | |  |
| 2'-O-methyl modified miR-4291 inhibitor AGCUGUUCCUGCUGAA | | |  |  | |  |
| miR-4291 for RT-PCR GTCGTATCCAGTGCAGGGTCCGAGGTATTCGCACTGGATACGACAGCTG | | |  |  | |  |
| qPCR for miR-4291 GCCGTTCAGCAGGAA GTGCAGGGTCCGAGGT | | |  |  | |  |
| qPCR for circLMO1 (Divergent primers) | CGTGTATCACCTCGACTGCT | GGTCCTTGATCTTGCGGTTA |  |  | |  |
| Convergent primers | CGTCCAGCCCAAAGGGAAG | GGCAGTCACAGCAGGCACAC |  |  | |  |
| qPCR for DHX9 | AGTCCTGTGCCCTGTCACTTGTC | GCTCCACTGTCTCTCCTTCCTTC |  |  | |  |
| qPCR for I1RC | GCCTATAATCCCAGCACTTTGG | TTTGGTAGAGACGGGGTTTCAC |  |  | |  |
| qPCR for I3RC | GTGATCTCGGCTCACTGCAAC | GTGAAACCCCATCTCTACTAAAAA |  |  | |  |
| qPCR for β-actin | GGCATCCACGAAACTACATTCAA | AGCCAGAGCAGTGATCTCCTTCT |  |  | |  |
| qPCR for GPX4 | CTTCACCAAGTTCCTCATCGACA | GTGGGGCAGGTCCTTCTCTAT |  |  | |  |
| qPCR for Acsl4 | TATTCTTCTCCGCTTACACTCTCTG | TATAATTCATCTCTTGGACTTTGCTC |  |  | |  |
| qPCR for Ptgs2 | ACCGAGGTGTATGTATGAGTGTGG | CCCTTGAAGTGGGTAAGTATGTAGTG |  |  | |  |
| qPCR for Nox1 | GTCACCCCCTTTGCTTCTATCTT | CTTTGCCTAATTCCTCCATCTCC |  |  | |  |
| qPCR for Slc7a11 | TGTCCGCAAGCACACTCCTC | CAGCAACTGCCAGCCCAAT |  |  | |  |
| qPCR for FTH1 | GCTCTACGCCTCCTACGTTTACC | TTTCTCAGCATGTTCCCTCTCC |  |  | |  |
| qPCR for Slc3a2 | GGGGACTAACTCCTCCGACCT | GAGCCTTGCCTGAGACAAACT |  |  | |  |
| pGL3-Acsl4-3’UTR | AGATATAGAGTCATTGTAATGTTG | TACAAATGTAATTTAATACATGTAC |  |  | |  |
| pGL3-circLMO1-wt | GCGTGCCGATGCTCTCCGTCCAG | CTCAGGTAGTCGCGTCGGCACAG |  |  | |  |
| siRNA for circLMO1 1# | [CCAGAGGCGTGCCGATGCTCT](http://blast.ncbi.nlm.nih.gov/Blast.cgi?PROGRAM=blastn&PAGE_TYPE=BlastSearch&LINK_LOC=blasthome&QUERY=%3Ehsa_circ_0021087-siRNA2%0ACCAGAGGCGTGCCGATGCTCT&DATABASE=nr&EQ_MENU=Homo%C2%A0sapiens%C2%A0(taxid:9606))dTdT | AGAGCATCGGCACGCCTCTGGdTdT |  |  | |  |
| siRNA for circLMO1 2# | [AACCAGAGGCGTGCCGATGCT](http://blast.ncbi.nlm.nih.gov/Blast.cgi?PROGRAM=blastn&PAGE_TYPE=BlastSearch&LINK_LOC=blasthome&QUERY=%3Ehsa_circ_0021087-siRNA1%0AAACCAGAGGCGTGCCGATGCT&DATABASE=nr&EQ_MENU=Homo%C2%A0sapiens%C2%A0(taxid:9606))dTdT | AGCATCGGCACGCCTCTGGTTdTdT |  |  | |  |
| Negative control siRNA | TTCTCCGAACGUGTCACGTdTdT | ACGTGACACGTTCGGAGAAdTdT |  |  | |  |
| siRNA for Acsl4 | TTCTGTTTCGTCTTCTTCTAdTdT | TAGAAGAAGACGAAACAGAAdTdT |  |  | |  |
| siRNA for DHX9 | TTGCAGAAATGACCATTTATAdTdT | TATAAATGGTCATTTCTGCAAdTdT |  |  | |  |

**Supporting Table S1. Sequence of primers, miRNA, and siRNA used in the study**

|  |  |
| --- | --- |
