# Supplementary material for: Circular RNA circLMO1 Suppresses Cervical Cancer Growth and Metastasis by Triggering miR-4291/ACSL4-Mediated Ferroptosis
Source: Front Oncol. 2022 Mar 7;12:858598. doi: 10.3389/fonc.2022.858598 (PMC8936435; doi:10.3389/fonc.2022.858598)
Supplement: Supplementary file 6 [file DataSheet_2.docx]

| **Target Detail** | **Target Rank** | **Target Score** | **miRNA Name** | **Gene Symbol** |
| --- | --- | --- | --- | --- |
|  | 1 | 83 | [hsa-miR-4291](http://mirdb.org/cgi-bin/mature_mir.cgi?name=hsa-miR-4291) | submission |
|  | 2 | 79 | [hsa-miR-4492](http://mirdb.org/cgi-bin/mature_mir.cgi?name=hsa-miR-4492) | submission |
|  | 3 | 74 | [hsa-miR-4498](http://mirdb.org/cgi-bin/mature_mir.cgi?name=hsa-miR-4498) | submission |
|  | 4 | 66 | [hsa-miR-762](http://mirdb.org/cgi-bin/mature_mir.cgi?name=hsa-miR-762) | submission |
|  | 5 | 66 | [hsa-miR-5001-5p](http://mirdb.org/cgi-bin/mature_mir.cgi?name=hsa-miR-5001-5p) | submission |
|  | 6 | 59 | [hsa-miR-922](http://mirdb.org/cgi-bin/mature_mir.cgi?name=hsa-miR-922) | submission |
|  | 7 | 58 | [hsa-miR-3085-5p](http://mirdb.org/cgi-bin/mature_mir.cgi?name=hsa-miR-3085-5p) | submission |
|  | 8 | 56 | [hsa-miR-6823-3p](http://mirdb.org/cgi-bin/mature_mir.cgi?name=hsa-miR-6823-3p) | submission |
|  | 9 | 56 | [hsa-miR-2114-3p](http://mirdb.org/cgi-bin/mature_mir.cgi?name=hsa-miR-2114-3p) | submission |
|  | 10 | 52 | [hsa-miR-3678-3p](http://mirdb.org/cgi-bin/mature_mir.cgi?name=hsa-miR-3678-3p) | submission |
|  | 11 | 52 | [hsa-miR-1827](http://mirdb.org/cgi-bin/mature_mir.cgi?name=hsa-miR-1827) | submission |
|  | 12 | 52 | [hsa-miR-4297](http://mirdb.org/cgi-bin/mature_mir.cgi?name=hsa-miR-4297) | submission |

**Supporting Table S2. The prediction of miRNA interacted with circLMO1 using miRDB (http://mirdb.org/mirdb/index.html).**
